# Complex Interplay between DNA Damage and Autophagy in Disease and Therapy

**DOI:** 10.3390/biom14080922

**Published:** 2024-07-29

**Authors:** Aman Singh, Naresh Ravendranathan, Jefferson C. Frisbee, Krishna K. Singh

**Affiliations:** 1 Department of Medical Biophysics, Schulich School of Medicine and Dentistry, University of Western Ontario, 1151 Richmond Street North, London, ON N6A 5C1, Canada; asing945@uwo.ca (A.S.); nravendr@uwo.ca (N.R.); jfrisbee@uwo.ca (J.C.F.); 2 Anatomy and Cell Biology, Schulich School of Medicine and Dentistry, University of Western Ontario, London, ON N6A 5C1, Canada

**Keywords:** autophagy, cancer, doxorubicin, BRCA1/2, DNA damage

## Abstract

Cancer, a multifactorial disease characterized by uncontrolled cellular proliferation, remains a global health challenge with significant morbidity and mortality. Genomic and molecular aberrations, coupled with environmental factors, contribute to its heterogeneity and complexity. Chemotherapeutic agents like doxorubicin (Dox) have shown efficacy against various cancers but are hindered by dose-dependent cytotoxicity, particularly on vital organs like the heart and brain. Autophagy, a cellular process involved in self-degradation and recycling, emerges as a promising therapeutic target in cancer therapy and neurodegenerative diseases. Dysregulation of autophagy contributes to cancer progression and drug resistance, while its modulation holds the potential to enhance treatment outcomes and mitigate adverse effects. Additionally, emerging evidence suggests a potential link between autophagy, DNA damage, and caretaker breast cancer genes BRCA1/2, highlighting the interplay between DNA repair mechanisms and cellular homeostasis. This review explores the intricate relationship between cancer, Dox-induced cytotoxicity, autophagy modulation, and the potential implications of autophagy in DNA damage repair pathways, particularly in the context of BRCA1/2 mutations.

## 1. Introduction

**Figure 1:**
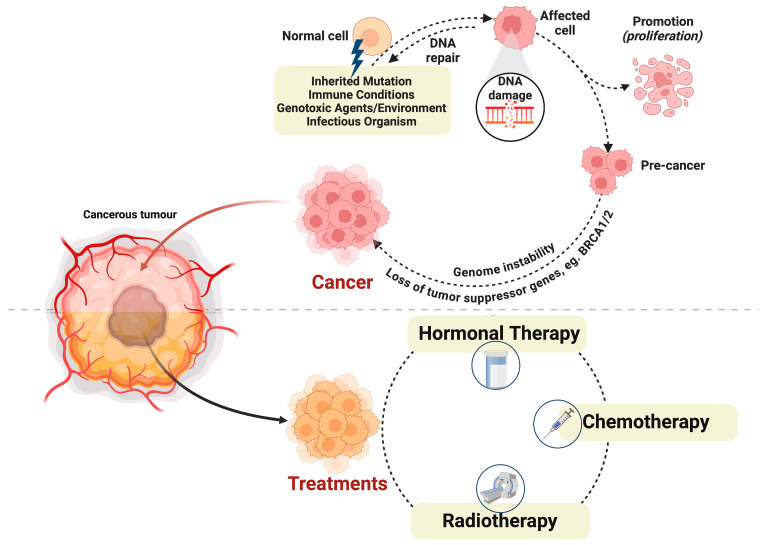
Cancer progression and treatment options. The figure illustrates the progression from normal to cancerous cells, detailing the factors contributing to DNA damage and subsequent cancer development. Inherited mutations, immune disorders, genotoxic agents, environment such as radiation, and infectious agents are shown as sources of DNA damage, initiating the formation of precancerous cells and promoting their proliferation. Key drivers like genomic instability and the inactivation of tumor suppressor genes, such as BRCA1 and BRCA2 (BRCA1/2), are depicted as crucial in cancer initiation. The figure also outlines cancer treatment options, including hormonal therapy, chemotherapy, and radiotherapy. *BRCA1/2: Breast Cancer Susceptibility Gene-1 and -2*. Created with Biorender.com.

Cancer, a complex and heterogeneous disease, remains a significant global health challenge characterized by uncontrolled cellular proliferation and growth due to genomic and molecular aberrations [1]. Cancer cells evade immune surveillance and exploit neighboring cells’ physiology to sustain their proliferation [2]. The metabolic mechanisms of cancer cells significantly overlap with those of host cells, posing a formidable challenge to cancer treatment [3]. In 2022 alone, the World Health Organization (WHO) reported a staggering 20 million new cancer cases worldwide, accompanied by 9.7 million cancer-related deaths, with projections indicating a rise to 35 million cases by 2050 [4]. Among the myriad cancer types, breast cancer ranks as the most prevalent [4]. These cancers arise from a combination of internal factors, such as inherited mutations and external factors acquired from the environment and infectious organisms [5] (Figure 1). Notably, DNA damage emerges as a critical player in cancer development, stemming mainly from mutations due to exposure to radiation or genotoxic agents or errors in DNA repair mechanisms triggering mutations or chromosomal instability, impacting crucial genes like oncogenes and tumor suppressor genes [6].

DNA damage can arise from various endogenous and exogenous sources, including oxidative stress, UV radiation, and genotoxic chemicals [7]. It represents a critical challenge to cellular integrity and genomic stability, necessitating intricate repair mechanisms to maintain genetic material. DNA damage encompasses a spectrum of lesions, ranging from single-strand breaks (SSBs) to double-strand breaks (DSBs) and base modifications. These lesions can disrupt normal DNA structure and function, potentially leading to mutations or genomic instability if left unrepaired. Cells have evolved a sophisticated network of DNA repair pathways, including base excision repair (BER), nucleotide excision repair (NER), mismatch repair (MMR), and homologous recombination (HR), among others, to mitigate the detrimental effects of DNA damage [8]. When DNA lesions persist without repair, prolonged DNA damage initiates specific cellular responses, including cell death and senescence. *Breast cancer susceptibility gene 1* (BRCA1) and *breast cancer susceptibility gene 2* (BRCA2) are critical components in maintaining genomic stability through their roles in the homologous recombination (HR) pathway. BRCA1 acts as a mediator and regulator of HR, facilitating the repair of DSBs [9]. Similarly, BRCA2 plays a key role in HR by aiding in the loading of RAD51 onto single-stranded DNA, crucial for strand invasion and repair of DSBs [10]. Mutations in BRCA1 or BRCA2 can lead to defective HR function, resulting in an accumulation of unrepaired DNA damage. This heightened genomic instability increases the risk of cancer development, particularly in breast and ovarian tissues where BRCA mutations are prevalent. A notable consequence of BRCA1/2 mutations is their impact on DNA repair efficiency and genome integrity. Cells with defective BRCA1 or BRCA2 are unable to effectively repair DNA damage, leading to the accumulation of lethal amounts of damaged DNA and spontaneous chromosomal abnormalities. This compromised DNA repair capacity predisposes cells to malignant transformation or apoptosis, highlighting the critical role of BRCA1 and BRCA2 in DNA damage repair in maintaining cellular health and preventing cancer [11,12,13,14]. A detailed review of BRCA1/2′s role in DNA damage repair is available elsewhere [15].

Although the world is still in search for an ideal therapy for cancer, the current therapies in use include surgery, radiation, hormonal and/or chemotherapy, and immunotherapy depending on the type, stage, and location of the cancer. Among these therapies, radiation and chemotherapy are discussed in detail here as these are the most common therapies and they exert their effect by inducing DNA damage in tumor cells. Details on hormonal and immunotherapies are provided elsewhere [16,17].

Radiation therapy stands as a cornerstone in the arsenal against cancer, harnessing the power of high-ionizing X-ray and gamma radiation to target and annihilate cancerous cells by inducing DNA damage and impeding their growth leading to cell death [18]. Radiation therapy plays a pivotal role in the treatment journey of over half of all cancer patients [19,20]. This therapeutic modality, however, confronts significant challenges as it unavoidably affects both malignant and healthy cells exposed to radiation. Radiation therapy comprises two primary modalities—(1) external beam radiation and (2) internal radiation or brachytherapy. External beam radiation therapy is the most prevalent form that employs a machine external to the body to deliver targeted radiation to the cancer site. Conversely, internal radiation therapy involves the placement of radioactive material directly into or near the tumor. This is particularly vital for patients with inoperable tumors or incomplete surgical resections, as well as those facing recurrent disease. Both are employed prophylactically to forestall recurrence and palliatively to alleviate symptoms of tumor progression and metastases, particularly in breast cancer treatment [21]. Furthermore, radiation therapy synergizes effectively with other modalities, such as chemotherapy and surgery [22,23,24]. However, despite its efficacy in combating cancer, concerns regarding its adverse effects on exposed healthy neighboring cells have been raised, and an increased risk of cardiovascular diseases has been documented [25,26]. Additionally, there exists a heightened risk of developing subsequent cancers following radiotherapy, including secondary malignancies [27]. These concerns underscore the ongoing imperative to refine and optimize radiation therapy protocols to minimize collateral damage while maximizing therapeutic efficacy in the ongoing battle against cancer.

Chemotherapy, which exerts its effect mainly by directly or indirectly targeting DNA, is widely used to treat different types of cancer, particularly advanced-stage breast cancer where the triple-negative subtype accounts for a notable 15–20% of all breast cancer cases [28]. Chemotherapeutic agents are categorized based on their mechanism of action and chemical structure, including alkylating agents and topoisomerase inhibitors [29]. Alkylating agents, for instance, disrupt DNA integrity by forming covalent adducts, hindering cell division, and inducing senescence. They interact with cellular DNA in their electrophilic forms, causing cross-linking of nucleic acids with proteins or peptides, leading to erroneous base pairing and DNA strand breakage [30]. This mechanism underlies their broader utility as frontline chemotherapeutic agents, particularly effective against slow-growing cancers. On the other hand, topoisomerase enzymes regulate DNA topology by facilitating the proper unwinding, separation, and rejoining of DNA strands during DNA replication and transcription process [31], and any dysregulation in their activity can lead to genomic instability and cancer. Thus, topoisomerase inhibitors act as anticancer agents and have emerged as a potential anticancer medication [31]. Inhibitors of DNA topoisomerase I and II exert their effects in cancer treatment. Type I inhibitors stabilize the cleavage complex, whereas type II inhibitors induce breaks in the complex along with DNA. These inhibitors demonstrate effectiveness in treating diverse cancers, including breast, lung, and ovarian cancers [31]. Chemotherapy can be administered intravenously, intramuscularly, intra-abdominally, topically, or through pressurized intraperitoneal aerosol chemotherapy, which is a new method for efficiently delivering intraperitoneal chemotherapy to patients with end-stage peritoneal metastases [32]. There are new therapeutic strategies on the rise that comprise small molecule inhibitors [33], amalgamations of anticancer agents coupled with immunotherapy [34], drugs utilizing nanotechnology and organo-seleno compounds [35,36]. Despite extensive research, targeted chemotherapy where the drug only reaches the tumor but not the healthy tissue is not established and, accordingly, poses significant challenges due to its adverse effects [37]. These include bone marrow injury [38], thrombocytopenia [39], substantial hepatotoxicity and nephrotoxicity [40,41], cardiotoxicity [42], and neurotoxicity [43]. Doxorubicin (Dox) exerts its effect mainly by inducing DNA damage and is renowned for its efficacy against various cancers, including breast, lung, leukemia, and carcinoma. However, its use is associated with significant drawbacks [44]. A detailed review comprising mechanistic details on Dox-induced DNA damage is available elsewhere [15]. Dox is derived from *Streptomyces bacteria* and typically administered intravenously at 21-day intervals with a dose ranging from 50 to 75 mg/m² [45]. One of the most concerning issues is its dose-dependent cytotoxicity on the heart and other organs, which has been the subject of an ongoing inquiry [10,42,46]. Administered in cancer therapy, Dox can precipitate cardiotoxic effects, potentially leading to heart failure [42,47]. Additionally, Dox’s neurotoxic effects can impair cognitive function, contributing to conditions called chemo-brain [43,46] and brain senescence [48]. Considering these adverse effects, optimizing therapeutic outcomes in cancer treatment requires vigilant monitoring and personalized approaches. The Dox’s cardiotoxic effect is such that in many of the cases, the treatment is halted to protect the heart regardless of the cancer status.

## 2. Doxorubicin-Induced Cardiotoxicity

Research on Dox-induced cardiotoxicity spans several decades. In the 1970s, Von Hoff and colleagues conducted early studies on 4018 patients confirming Dox-induced congestive heart failure (CHF) and its relationship with the cumulative dose of administered Dox, with a continuum of increasing risk with dose and/or age. Surprisingly, their research also revealed that a weekly dosing schedule of Dox was linked to a significantly lower incidence of CHF compared with the standard three-week schedule, challenging prevailing understandings of cumulative dose-related toxicity [45,49]. Throughout the 1980s and 1990s, investigations focused on dose dependency and risk factors in Dox-induced CHF [50]. By the late 1990s, Dox’s clinical use was limited by dose-related cardiomyopathy [51]. Further, histological evaluation in both human and animal models’ hearts revealed characteristic myocardial changes, such as myocardial fibrosis, myofibrillar disarray, and vacuolar degeneration of cardiomyocytes associated with Dox-induced cardiotoxicity [52]. Moreover, the incidence of Dox-induced cardiomyopathy significantly escalates beyond a cumulative dose of 550 mg/m^2 body surface area. Consequently, once this threshold is surpassed, the therapy is typically omitted from chemotherapy regimens [53]. This outcome underscores the potential deprivation of patients from an effective treatment option to mitigate Dox’s cardiotoxic side effects.

**Figure 2:**
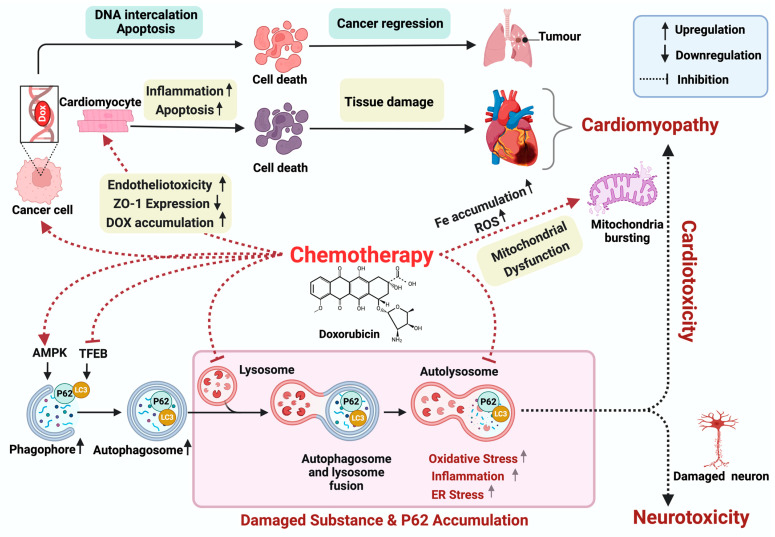
Chemotherapy-induced toxicity. This figure elucidates the repercussions of chemotherapeutic drugs on neuronal and cardiac systems. Specifically, doxorubicin (Dox) is portrayed as an agent that inhibits TFEB activity while stimulating AMPK, thereby fostering the upregulation of phagophore and autophagosome formation. However, Dox concurrently hampers lysosomal function and autophagosome maturation, leading to the accumulation of damaged substrates and p62. This accumulation exacerbates oxidative stress, inflammation, ER stress, and mitochondrial dysfunction, culminating in neuronal damage, thereby inciting neurotoxicity and contributing to cardiotoxicity. Additionally, Dox intercalates with DNA in cancer cells, leading to apoptosis and regression of cancer. Dox-induced cardiotoxicity primarily arises from mitochondrial dysfunction, characterized by elevated iron accumulation and increased reactive oxygen species generation. Dox promotes tissue permeability by inducing endothelial toxicity and inhibiting ZO-1 expression, which intensifies drug retention within cardiac tissues. Consequently, this provokes inflammation, apoptosis, tissue degeneration, and, ultimately, cardiomyopathy. The figure illustrates how the inhibition of autophagosome and lysosome maturation by Dox exacerbates cellular damage and dysfunction, amplifying the pathogenesis of neurotoxicity and cardiotoxicity. The accumulation of damaged substances and p62 further highlights the dysregulation of autophagy and its contribution to cellular pathology, including mitochondrial dysfunction, which further exacerbates cardiotoxicity. *Dox: Doxorubicin, ROS: Reactive Oxygen Species, p62: Sequestosome 1, TFEB: Transcription Factor EB, AMPK: AMP-activated Protein Kinase, ZO-1: Zonula Occludens-1*. Created with Biorender.com.

In the 2000s, research shifted toward understanding the mechanism of Dox-induced cytotoxicity and developing a personalized approach to mitigating this risk. The pathogenesis of Dox-induced cardiac dysfunction remains unclear; however, oxidative stress, apoptosis, and mitochondrial dysfunction are implicated [15]. This is characterized by an imbalance in reactive oxygen species (ROS) and reactive nitrogen species (RNS) causing dysregulated antioxidant mechanisms resulting in subcellular damage and apoptosis [15]. Additionally, studies on murine models have shown that Dox-induced cardiotoxicity primarily arises from oxidative stress and mitochondrial dysfunction, characterized by reduced oxygen consumption, decreased mitochondrial membrane potential, and elevated iron accumulation [54] (Figure 2). The investigation conducted by Pillai et al. reveals that the decline in SIRT3 levels, a mitochondrial enzyme that regulates cellular metabolism and oxidative stress response, which is crucial for maintaining mitochondrial function, correlates with the cardiotoxic repercussions of Dox [55]. Moreover, Jordan et al.’s findings in 2018 highlight the frequent occurrence of cardiac atrophy in patients subjected to Dox therapy [56]. Using an in vivo study, Ni et al. uncovered additional factors contributing to the adverse effects of Dox demonstrating its ability to induce inflammation in a more intricate manner. Notably, beyond stimulating CD11b+ macrophages to produce IFNγ, Dox was found to disrupt lipid metabolism, presenting a multifaceted mechanism underlying the development of cardiomyopathy [57]. In the presence of iron (Fe), Dox undergoes futile redox cycling, generating ROS and causing cellular damage. This process involves superoxide formation, conversion to hydrogen peroxide (H2O2), and production of highly toxic hydroxyl radicals through the Fenton reaction. Additionally, Dox directly interacts with Fe to form a Fe–Dox complex, further enhancing ROS production [58]. In the other study, Liu et al. (2018) observed that Dox also disrupts AMPK function and suppresses PGC1-α. This disruption alters downstream antioxidant signaling pathways involving NRF1, TFAM, and UCP2. Consequently, cardiomyocyte viability is compromised, which exacerbates cardiac injury [59]. Dox binding to eNOS reductase causes an imbalance between nitric oxide and superoxide, leading to cardiotoxicity via increased redox stimulation, and apoptosis [60,61].

Importantly, Dox disrupts the crucial endothelial cell–cardiomyocyte bond, exacerbating cardiac injury by enhancing ROS-driven damage, reducing zonula occludens-1 (ZO-1) expression, and increasing permeability, while also heightening Dox accumulation in the heart [15,62]. Furthermore, Dox-induced ROS and RNS production induce mitochondrial damage in endothelial cells, triggering apoptosis through cytochrome C release and caspase activation, disrupting their supportive role for cardiomyocytes [63]. Moreover, studies on cultured endothelial cells reveal ROS/RNS-independent direct DNA damage by Dox binding with CG-rich sequences, further disrupting endothelial–cardiomyocyte crosstalk and support [64]. Furthermore, exposure to Dox during the early stages of life in mice led to a failure in developing compensatory cardiac hypertrophy when later challenged with angiotensin-II (Ang-II)-induced hypertension [65]. This suggests that the cardiotoxic effects of Dox may interfere with the heart’s ability to adapt to increased blood pressure, potentially exacerbating the detrimental effects of hypertension on cardiac function. The extensive research on Dox-induced cardiotoxicity underscores its complex pathogenesis and multifaceted impact on cardiac function. From historical observations to contemporary insights, evidence highlights its detrimental effects, including dose-dependent cardiomyopathy, CHF, and disruption of cell interactions. Understanding these mechanisms and developing personalized approaches are crucial for mitigating risks and improving outcomes.

## 3. Doxorubicin-Induced Neurotoxicity

Chemotherapy-induced cognitive decline, often termed chemotherapy-related cognitive impairment (CRCI) or “chemo-brain”, is a recognized phenomenon observed in cancer patients undergoing Dox chemotherapy [46]. Manifesting as cognitive difficulties, this condition affects a significant proportion, approximately 17–70% of cancer patients, and is an outcome of a complex interplay of mechanisms [66]. These include the induction of DNA damage [67], augmentation of oxidative stress pathways [68,69], initiation of inflammatory cascades [70,71], disruption of apoptosis, modulation of neurotransmitter levels [72], perturbation of mitochondrial function [73], and inhibition of neurogenesis [74]. In the 1980s, there was a prevalent notion that Dox did not pose a risk of neurotoxicity due to its limited penetration of the blood–brain barrier (BBB) [75]. Nevertheless, doubts regarding the potential for Dox-induced neurotoxicity persisted. Thus, multiple studies conducted in the 2000s have suggested a potential indirect mechanism, which is Dox-induced elevated levels of peripheral TNF-α that may traverse the blood–brain barrier (BBB) and impede cellular antioxidant systems [76,77] . In a separate investigation conducted in mice, it was found that exposure to Dox induces significant mitochondrial damage and disrupts the levels of brain choline-containing metabolites and phospholipases, and the shifts in metabolic markers are closely associated with heightened oxidative stress mediated by TNF-α [73]. Indirect Dox-induced neurotoxicity by TNF-α, which has the potential to influence the volume of the hippocampus, can augment the inflammation by activating astrocytes and microglia in the brain [70,71]. Additionally, animal studies witnessed that Dox treatment significantly reduces neurogenesis, evidenced by a notable decrease in the number of cells labeled with the neuro-specific nuclear antigen bromodeoxyuridine (BrdUrd), which are crucial for spatial processing and memory formation [74]. In our current mice study, we observed increased DNA damage measured by immunoblotting for gH2AX—a marker for DNA damage—showing the potential role of DNA damage in Dox-induced neurotoxicity (*unpublished data*); however, it remains to be confirmed whether it is a direct or indirect effect of Dox. Among clinical observations spanning the past two decades, Freeman and Broshek (2002) elucidated the cognitive impairments associated with Dox-based chemotherapy, ranging from memory deficits to depressive symptoms in breast cancer patients [78]. Building upon these findings, studies in 2016 delineated a spectrum of neurological symptoms, including headaches, seizures, and encephalopathy, predominantly observed in patients treated with Dox [79,80]. In 2017, Cruz-Carreras et al. reported specific neurological symptoms, such as headaches and aphasia, in a young leukemia patient undergoing Dox chemotherapy. Additionally, these patients reported decreased strength in the right arm, which shows a potential connection with the central nervous system and the peripheral nervous system [81]. Most recently, a study illuminated the prevalence of posterior reversible encephalopathy syndrome (PRES) in children undergoing chemotherapy, with seizures emerging as a prominent clinical feature [82]. Indirect neurotoxicity resulting from Dox treatment is attributed to the induction of oxidative stress due to excessive production of ROS [69]. These ROS subsequently lead to the oxidative modification of proteins, lipids, and nucleic acids. Additionally, Hsieh et al. demonstrated that ROS could activate nuclear factor kappa B (NF-κB), further implicating oxidative stress in the neurotoxic effects of Dox [83]. The neurological impact of Dox is complex, necessitating further investigation crucial for optimizing therapies and minimizing adverse effects to improve patient outcomes. Current drug discovery focuses on novel derivatives with enhanced efficacy and reduced toxicity, including natural compounds from microbial, plant, and Ayurvedic sources [84,85,86].

Despite extensive investigation around the role and contribution of DNA damage to Dox-induced toxicity, there still exists a gap in knowledge about the role of different mechanisms. Recently, autophagy has emerged as a crucial pathway in both cancer and healthy cells that are exposed to Dox. Consequently, there has been a recent shift in research focus from DNA damage to autophagy in Dox-associated toxicity. While chemotherapy remains integral to cancer treatment, this review will concentrate on elucidating the adverse DNA damage effects induced by Dox and its direct and indirect intricate relationship with autophagy in disease and cancer. By delving into the complex pathways of autophagy and its multifaceted roles, this review also aims to highlight the potential of various factors in modulating autophagy to mitigate Dox-induced adverse effects, thereby offering promising avenues for therapeutic intervention.

## 4. Autophagy

**Figure 3:**
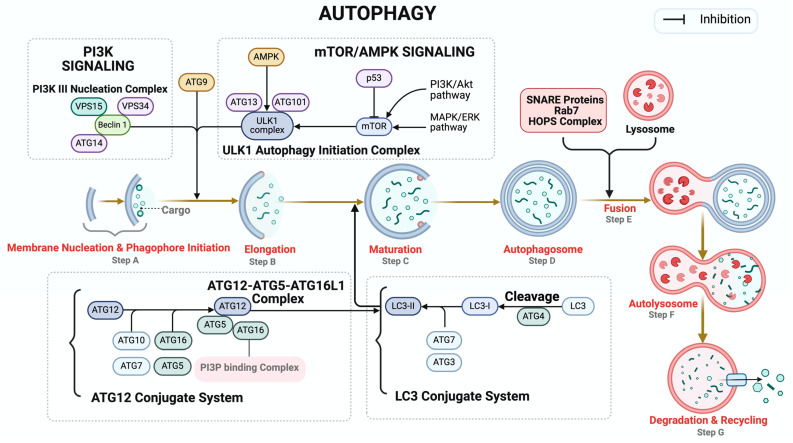
Autophagy process and protein complexes. Autophagy, a complex cellular degradation process, progresses through initiation, elongation, maturation, fusion, and degradation phases. Its regulation involves mTOR inhibition and AMPK activation, while autophagy-related proteins (ATGs) such as VPS15 and VPS34 and BECN1 are essential for its execution. In cancer, p53 inhibits mTOR, which is a negative regulator of autophagy, thus exacerbating autophagy for cancer cells’ survival under stress. When autophagy is initiated, unwanted cytoplasmic materials (autophagic cargo) are engulfed by a double membrane, signaling the beginning of the cleaning process (Step A). Next, the cell forms a structure called the phagophore, akin to preparing a basket to collect items for cleaning (Step A). After the phagophore expands to form the autophagosome, it increases its capacity to hold more cellular components slated for recycling (Step B, Step C, Step D). Subsequently, the autophagosome fuses with acidic lysosomes (Step E) to form autolysosomes, wherein SNARE proteins, the HOPS complex, and Rab7 play crucial roles in mediating the fusion between autophagosomes and lysosomes, facilitating cargo degradation within autolysosomes (Step F). These cellular organelles are responsible for degradation and recycling, facilitating the breakdown of the collected material into reusable components (Step G). Central to the orchestration of autophagy are various protein complexes: the ULK1, an autophagy initiation complex, kickstarts the process; the nucleation complex and PI3k-binding complex aid in the formation and guidance of the phagophore akin to architectural blueprints and construction crews assembling a structure. ATG9-containing vesicles, along with proteins like VPS15 and VPS34, support phagophore expansion by generating PI3P on membranes, recruiting necessary components. In the ATG12 conjugation system, ATG12 is conjugated with ATG5 to form a complex with ATG16L1. This complex then interact with the PI3P-binding complex, which is involved in phagophore elongation. The ATG12–ATG5–ATG16L1 complex also promotes conjugation of LC3, whereby LC3 is cleaved by the protease ATG4 to form LC3-I, which is then conjugated to form LC3-II. Once LC3 conjugation is complete, it assists in forming a mature phagophore by extending the phagophore. Then the autophagosome fuses with a lysosome to form the autolysosome and degrade the contents. ATG9 vesicles supply membrane material, ensuring efficient cellular maintenance. *AMPK: AMP-activated protein kinase, ATG: autophagy-related proteins, mTOR: mechanistic target of rapamycin, BECN1: Beclin1, VPS34/15: vesicular protein sorting 34/15, PI3P: phosphatidylinositol 3-phosphate, PE: phosphatidylethanolamine, SNARE: soluble N-ethylmaleimide-sensitive factor attachment protein receptor, HOPS: homotypic fusion and protein sorting, Rab7: ras-related protein Rab-7, WIPIs: WD repeat domain phosphoinositide-interacting proteins, DFCP1: zinc-finger FYVE domain-containing protein 1, LC3: microtubule-associated protein light chain 3, and ULK: unc-51-like autophagy activating kinase 1*. Created with Biorender.com.

Autophagy is a highly conserved cellular process involved in the degradation and recycling of damaged organelles and proteins, thus playing a crucial role in maintaining cellular homeostasis [87]. Dysregulation of autophagy has been implicated in various pathological conditions, including cardiovascular diseases [88,89,90], neurodegenerative diseases [91], cancer [92], metabolic disorders [93], and infectious diseases [94]. This tightly regulated process culminates in the formation of autophagosomes, which are double-membraned vesicles that ultimately fuse with lysosomes. This fusion allows for the degradation of protein aggregates and dysfunctional organelles, promoting cell survival. The maturation of autophagosomes involves several coordinated steps, including initiation, elongation, maturation, fusion, and degradation—all tightly governed by signaling pathways and protein complexes to ensure an efficient and selective breakdown of cellular components [95] (Figure 3). Initiation begins with the formation of the phagophore, facilitated by the activation of Unc-51-like kinases (ULKs). The activity of ULK is modulated by mTORC1, which is a nutrient sensor. Under conditions of nutrient abundance, mTORC1 activation suppresses autophagy initiation. Conversely, during nutrient deprivation, autophagy is activated by the inhibition of the mTORC1 [96]. AMPK, an energy sensor, activates autophagy by inhibiting mTORC1 [97,98,99]. Autophagy process requires two ubiquitin-like conjugation systems: the ATG12-ATG5-ATG16L1 complex and the microtubule-associated protein 1A/1B-light chain 3 (LC3) system [100]. The ATG12-ATG5-ATG16L1 complex is crucial for the lipidation of LC3 as it covalently attaches ATG3 to LC3 [101]. LC3 is processed by ATG4 and conjugated to phosphatidylethanolamine (PE) to form LC3-II, which is integrated into the autophagosome membrane by the E3-like complex to support its expansion and closure [102]. During the maturation step, the original phagophore develops into a fully matured autophagosome, supported by the recruitment of more LC3-II and the closing of the phagophore to produce a double-membraned autophagosome, which then fuses with lysosome for degradation (Figure 3) [103].

The Beneficial and Detrimental Role of Autophagy in Cancer: Prior to delving into the role of autophagy in Dox-induced toxicity and discussing the possibility of modulating autophagy to reduce unwanted Dox-induced toxicity, it is imperative to understand the role of autophagy in cancer itself. In cancer, autophagy exhibits diverse and, sometimes, conflicting roles that are influenced by factors such as tissue type, tumor stage, and the microenvironment, necessitating a comprehensive understanding for the development of effective therapeutic strategies [104,105,106]. Autophagy can act as a tumor suppressor mechanism that inhibits tumor growth and as a cytoprotective mechanism that promotes tumor survival [107,108]. Autophagy functions as a tumor suppressor through a variety of methods. It removes defective organelles and misfolded proteins to prevent cellular harm, which reduces inflammation, a known risk factor for cancer growth [109], and minimizes the accumulation of mutations that could lead to tumor initiation [110]. For example, the tumor suppressor gene, *cation transport regulator homolog 2* (*CHAC2*), which is typically downregulated in gastric and colorectal cancer, is degraded by the ubiquitin–proteasome pathway. However, CHAC2 expression is essential to inhibiting tumor growth, proliferation, and migration, as CHAC2 induces mitochondrial apoptosis and autophagy [111]. Autophagy also reduces oxidative stress by digesting damaged mitochondria, a process known as mitophagy, which is a process of selectively targeting damaged mitochondria for degradation to regulate mitochondrial quality control [112]. Mitophagy is crucial to maintaining ROS levels, limiting DNA damage, and eventually preventing cancer [112]. By encouraging the elimination of damaged mitochondria and other cellular components, autophagy aids in preserving cellular energy balance and lowering oxidative stress [99,113].

Autophagy also serves as a pivotal mechanism in responding to cellular stress, capable of inducing cell cycle arrest and apoptosis. This response can potentially restrict the proliferation of cells carrying harmful mutations. Autophagy facilitates the degradation of key regulators of the cell cycle, such as cyclins and cyclin-dependent kinases, crucial for cell cycle progression. Notably, studies have demonstrated autophagic degradation of cyclin A and CDK2, effectively limiting the proliferation of mutation-bearing cells and impeding cancer progression [114,115]. Autophagy is also involved in the apoptotic pathway. Autophagy is used to degrade specific anti-apoptotic factors to promote the apoptotic pathway. Caspases are also activated by the degradation of caspase inhibitors or the release of pro-apoptotic factors from the mitochondria [116]. Several molecular pathways regulate the role of autophagy in cell cycle arrest and apoptosis. For example, the inhibition of mTOR induces autophagy. Research has demonstrated that substances such as apigetrin can cause cell cycle arrest and apoptosis through the PI3K/AKT/mTOR pathway, which, in turn, causes a decrease in cancer cell growth and an increase in cell death [117]. However, in established cancers, the observed detrimental role of autophagy promotes tumor survival, particularly under stressful conditions such as nutrient deprivation, hypoxia, and therapeutic stress [118]. In these contexts, autophagy recycles cellular components to provide a continuous source of energy and essential biomolecules to tumor cells, especially in nutrient-deprived conditions that support tumor growth and migration [106]. Additionally, autophagy helps cancerous cells adapt to different conditions, such as radiation and chemotherapy by degrading and recycling damaged cellular components, thereby enhancing cell survival under therapeutic stress and promoting therapeutic resistance [119]. Autophagy can influence the tumor microenvironment by regulating the availability of oxygen and degrading damaged organelles, which aids in the survival of cancer cells in hypoxic environments. In an unfavorable microenvironment, autophagic activity helps cancer cells survive and proliferate by recycling intracellular components to provide nutrition [105]. Autophagy also supports critical processes required for cancer spread, such as cell invasion and migration, by supplying energy and preserving cellular homeostasis that promotes metastasis.

Autophagy-based Clinical Trials in Cancer: Drugs that inhibit the late phases of autophagy, such as hydroxychloroquine (HCQ) and chloroquine (CQ), are being investigated to sensitize cancer cells to chemotherapy and other treatments [120]. Both CQ and HCQ inhibit autophagy by inhibiting autophagosome fusion with lysosomes, eventually dampening the growth of malignant cells. When the autophagy system is disrupted, defective autophagosomes accumulate resulting in cellular stress and, perhaps, leading to cancer cell death [121]. By increasing the susceptibility of cancer cells to treatment-induced cell death, this can improve the efficacy of other cancer therapies. These drugs are already approved for use in malaria and rheumatoid arthritis and are being repurposed for cancer therapy in various clinical trials [122]. However, the efficacy of treatment varies in different tissues due to different cancer lines relying on autophagy more than others. Clinical trials on CQ and HCQ have demonstrated their potential as useful adjuvant therapies in a variety of cancer treatments. In one trial, CQ and HCQ were used with antiestrogen treatments for estrogen receptor-positive (ER+) breast cancer, such as tamoxifen (TAM) and faslodex (ICI). The result demonstrated that CQ could reverse the resistance of breast cancer cells to estrogen. TAM and CQ demonstrated success collectively, indicating that CQ may improve the receptivity of ER+ breast cancer patients to antiestrogen treatments [123]. Another study investigated the treatment of glioblastoma using CQ in addition to radiation therapy, leading to an increase in radiation therapy’s efficacy, indicating that CQ may be a useful adjuvant therapy for treating glioblastoma [124]. Preclinical studies have also demonstrated HCQ’s efficacy in conjunction with temozolomide, a popular chemotherapeutic treatment for glioblastoma. The findings imply that the inhibition of autophagy by HCQ increases the susceptibility of glioblastoma cells to chemotherapy, which may improve tumor growth control and patient recovery [125]. In scenarios where enhancing autophagy may lead to cell death or prevent tumor initiation, activating autophagy could be beneficial. This approach could be particularly effective in early-stage cancers or as a preventive strategy in high-risk populations. Drugs like rapamycin and its analogs (temsirolimus, everolimus) inhibit the mTOR pathway, a negative regulator of autophagy [126]. These medications can cause death in cancer cells reliant on mTOR signaling by triggering autophagy. Treatments for certain diseases, including pancreatic neuroendocrine tumors, renal cell carcinoma, and breast cancer, involve the use of mTOR inhibitors, which may also improve the effectiveness of other cancer treatments [127]. Dox is the most common drug used in cancer chemotherapy and given the context-dependent complex, detrimental and beneficial, role of autophagy in different cancer types, it becomes imperative to characterize autophagy and its role for every cancer type before designing an autophagy-based therapy, such as Dox-based chemotherapy.

Doxorubicin Chemotherapy and Autophagy: Dox induces autophagy in various cancer cells. This autophagic reaction may function as a defense mechanism, enabling cancer cells to tolerate the damaging impacts of Dox. Conversely, Dox is also known to suppress autophagy, compromising DNA repair pathways and, potentially, inducing apoptosis in non-cancer cells, which contributes to dilated cardiomyopathy [128]. Several interrelated pathways, including mitochondrial damage and ROS production, forkhead box o3a (FOXO3a) and miR-223 regulation, PI3K/AKT/mTOR pathway, p53 pathway, and AMPK/mTOR signaling, play a role in the induction of autophagy by Dox [129,130,131]. These methods illustrate autophagy’s significance in cancer treatment by proving to be both a possible therapeutic target and a cell survival mechanism. Understanding these pathways can support the development of combination therapies that serve to manipulate autophagy and boost Dox’s efficacy while simultaneously minimizing its side effects. Dox-induced mitochondrial dysfunction can induce ROS production, which, in turn, promotes autophagy. Ozcan et al. highlighted this process in their discovery of higher levels of autophagic markers (LC3-II and Beclin-1) in cardiac tissues obtained from Dox-treated patients. This study demonstrated that the accumulation of damaged mitochondria and increased production of ROS were important driving factors of the autophagic response [130]. Dox treatment is also associated with an increased expression of ATGs such as ATG5 and Beclin-1, demonstrating elevated autophagic activity [129,132]. The conversion of LC3-I to LC3-II is an essential step in the development of autophagosomes [133]. Dox therapy raises LC3-II levels indicating increased autophagosome production, which is supported by the increase of autophagic vacuoles seen both in vitro and in vivo [129]. The transcription factor FOXO3a is a key regulator of autophagy. According to Zhou et al., hepatocellular carcinoma cells (HCCs) overexpress miR-223, leading to the downregulation of its target FOXO3a, which prevents Dox-induced autophagy and reverses chemoresistance in HCC cells [131]. One important regulator of cell proliferation, survival, and autophagy is the PI3K/AKT/mTOR pathway, and its inhibition activates autophagy. It has been shown that Dox interacts with this mechanism to induce autophagy. Moreover, natural substances such as magnoflorine have been shown to enhance Dox sensitivity by blocking the PI3K/AKT/mTOR pathway, thereby inducing autophagy and promoting cell death in breast cancer cells. This investigation demonstrated that the increased autophagic response displayed in Dox-treated cells is dependent on the inhibition of the PI3K/AKT/mTOR pathway [134]. Dox also activates the AMP-activated protein kinase (AMPK) pathway, which acts as an energy sensor in cells and suppresses the mTOR pathway and, thereby, activates autophagy in HCC cells [135]. Additionally, Ang-II, a key component of the renin-angiotensin system, is also reported to exacerbate Dox-induced cardiac injury through dysregulation of autophagy pathways [136].

**Figure 4:**
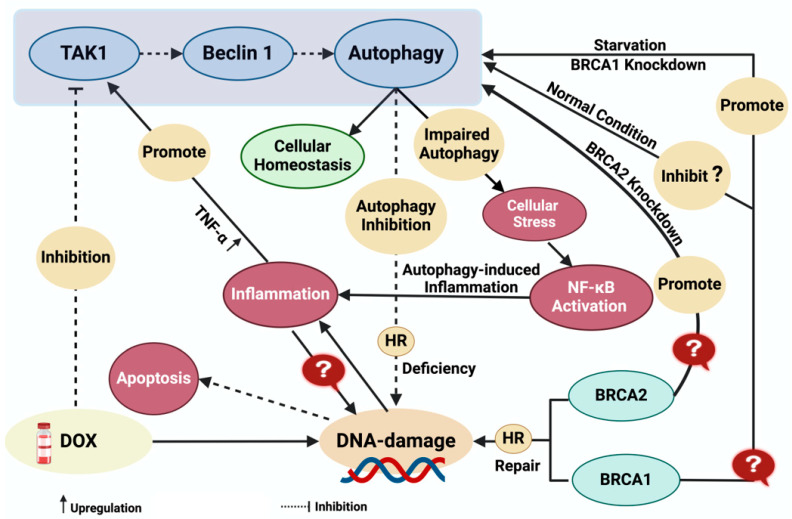
The interplay between DNA damage, inflammation, and autophagy in Doxorubicin-induced cellular responses: role of BRCA1/2. The figure presents a comprehensive overview of the cellular response to Dox-induced DNA damage, highlighting the intricate interplay between DNA damage repair, inflammation, BRCA1/2 genes, and autophagy-mediated cellular homeostasis. Dox-induced DNA damage triggers a cascade of events leading to the release of proinflammatory cytokines, such as TNF-α, which activate signaling pathways involving TAK1 and BECLIN, ultimately promoting autophagy. This autophagic response is crucial for mitigating inflammation and facilitating DNA damage repair; however, impaired/inhibited autophagy exacerbates cellular stress, promotes inflammation via NF-kB activation, and contributes to DNA damage accumulation. The complex crosstalk and the underlying mechanism between DNA damage and inflammation induced by autophagy are not clear, as shown by question mark sign. Additionally, Dox is also known to inhibit autophagy, which causes HR deficiency and excessive accumulation of DNA damage compromising genomic integrity. Notably, the figure also illustrates the involvement of key DNA damage repair proteins, BRCA1 and BRCA2, in the HR pathway. While BRCA negatively regulates autophagy, exhibiting an antioxidant role under starved conditions. Despite this understanding, the precise molecular mechanisms underlying BRCA modulation of autophagy remain elusive, as shown by question mark sign. The figure thus provides a comprehensive portrayal of the interconnected pathways governing cellular response to DNA damage; however, it also underscores the need for deeper mechanistic insights to fully elucidate the crosstalk between autophagy, DNA damage repair, and inflammation regulation, which could hold significant implications for therapeutic strategies targeting DNA damage-associated pathologies. *Dox: Doxorubicin, BRCA1/2: breast cancer type 1/2 susceptibility protein, TAK1: transforming growth factor beta-activated kinase 1, TNF-α: tumor necrosis factor-alpha, HR: homologous recombination*. Created with Biorender.com.

Genomic integrity is a fundamental aspect of cellular health and disease prevention, and DNA damage poses significant threats to genomic stability and is primarily caused by factors like ROS, replication stalling, and genotoxic or radiation exposure [137]. Autophagy, a critical cellular process responsible for clearing damaged components, has become recognized for its role in maintaining genomic stability and its relevance to conditions like breast cancer [138,139]. Previous investigations utilizing genetic inhibition of autophagy (via ATG7 knockout) in fibroblasts have revealed autophagy’s impact on DNA repair mechanisms, primarily homologous recombination (HR), leading to HR deficiency characterized by genomic instability and an increased apoptosis [140]. Persistent HR deficiency leading to accumulation of DNA damage can lead to the activation of inflammatory pathways, and recent studies have linked DNA damage and inflammation in the context of Dox-induced cardiomyopathy. Dox-induced DNA damage triggers inflammatory responses characterized by the upregulation of pro-inflammatory cytokines, including tumor necrosis factor-alpha (TNF-α), which, in turn, can activate autophagy-related pathways (Figure 4) [141,142]. Inflammation is a key factor in Dox-induced cardiotoxicity where Dox-induced cytokines like interleukin-1 and TNF-α trigger NF-κB and p38-MAPK signaling pathways leading to inflammatory cascades [143,144]. NF-κB, a central regulator of inflammation, is activated by the IκB kinase complex involving transforming growth factor-β (TGF-β)-activated kinase 1 and its cofactors TAK1 and MAP3K7-binding proteins. Once activated, NF-κB translocates to the nucleus to induce an inflammatory response [143,145]. Inflammatory and autophagy pathways are interwoven as certain components associated with TAK1, like TGF-β-activated kinase 1-binding proteins 2/3, can engage Beclin-1 to promote autophagy [146]. Reports indicate that compromised autophagy leading to p62 accumulation triggers IKK activation, subsequently inducing NF-κB-mediated inflammation; conversely, enhanced autophagy prevents p62 accumulation, thereby impeding the NF-κB signaling pathway [147]. However, the precise molecular mechanisms mediating the communication between genomic instability, inflammation, and autophagy pathways remain to be fully elucidated.

Dox-induced dysregulation of autophagy leads to the accumulation of autophagosomes, disrupting cellular health and therapeutic efficacy [148]. This dysregulation underscores the intricate relationship between autophagy, DNA repair, and disease processes as evidenced by promising pre-treatment strategies that enhance autophagy to mitigate Dox-induced cardiotoxicity [129]. The essential role of autophagy in maintaining genomic integrity is highlighted by perturbations in checkpoint kinase-1 (CHK1) regulation and degradation mechanisms [138]. However, paradoxically, Dox suppresses autophagy, compromising DNA repair pathways and, potentially, inducing apoptosis, which contributes to dilated cardiomyopathy [128]. The interplay between DNA damage and autophagy is heavily influenced by DNA-damage repair (DDR) genes, such as breast cancer susceptibility genes 1 and 2 (BRCA1/BRCA2), and Fanconi anemia (FA) genes. Cells lacking DDR genes, particularly BRCA genes, are particularly susceptible to Dox-induced toxicity, showing a 70-fold increase in vulnerability compared with normal cells [149]. This susceptibility underscores the critical role of DNA double-strand break (DSB) repair mechanisms orchestrated by BRCA1/BRCA2, as well as the active involvement of the FA-BRCA pathway in DDR [150,151,152,153]. Of note, the chromatin complex, involving monoubiquitinated FANCD2 (FANCD2-L) and BRCA2, is implicated in homology-directed repair (HDR) although the precise nature of their interaction remains unclear. BRCA2 actively engages with RAD51 to facilitate DNA damage repair, while FANCD2-L likely assists in ensuring precise matching of genetic material for effective RAD51 unloading by BRCA2 at targeted repair loci [151]. Mutations in DDR molecules, particularly FA genes (e.g., PALB2) and BRCA genes (e.g., BRCA1) can lead to significant complications, including breast/ovarian cancer and severe forms of Fanconi anemia [154,155,156,157]. Studies have elucidated the relationship between BRCA genes and autophagy, revealing that BRCA1-deficient cells heavily rely on autophagy for survival, with disruption of autophagy leading to rapid cell death [158]. Conversely, studies observed that BRCA1 negatively regulates autophagy in MCF-7 breast cancer cells, with BRCA1-loss increasing autophagic vacuoles and ROS levels. This suggests a potential antioxidant role of BRCA1, influencing heightened autophagy due to mitochondrial dysfunction and increased ROS levels [159]. Additionally, BRCA1 levels have been found to decrease rapidly during nutrient deprivation-induced autophagy in MCF-7 cells [159]. Loss of BRCA2 in tumors already exhibiting BRCA1 allelic loss enhances autophagy and mitophagy, suggesting a potential link between BRCA2 and autophagy [160]. However, the clear role of BRCA2 in regulating autophagy remains unclear. Autophagy is known to be essential for enabling HR, and its absence leads to DNA damage accumulation and cell death (Figure 4) [138]. Exploring the role of BRCA2 in these processes could offer crucial insights into DNA repair, genome stability, and disease development. Furthermore, understanding how autophagy is influenced in different BRCA gene variants and HR-deficient tumors, as well as how Dox affects autophagy in BRCA2-mutated patients, is pivotal for refining breast cancer treatment strategies. Singh et al. found that cardiomyocyte-specific BRCA1-deficient mice exhibited significant metabolic disruptions, including decreased expression of glucose and fatty acid transporters, along with reduced levels of key enzymes involved in fatty acid oxidation. This led to diminished rates of glucose and fatty acid oxidation despite increased activation of AMPK and AKT, indicating an energy-starved heart and predisposing the heart to failure [161]. There is also a growing interest in understanding how BRCA genes responsible for DNA damage, known for their roles in cancer and immune response, may regulate autophagy, a process critical for cardiac homeostasis. However, the specific interaction between BRCA1 and autophagy pathways in the context of cardiac metabolism and heart failure remains unclear. Further investigation is essential to elucidating this potential interaction and its implications for therapeutic intervention [159,162].

## 5. Doxorubicin and Mitophagy

Mitochondria, often referred to as the powerhouse of the cell, are vital for maintaining genomic integrity alongside DDR genes [163]. Unlike nuclear DNA, mitochondrial DNA (mtDNA) is highly susceptible to damage, posing significant risks to cellular health and contributing to various diseases, such as neurodegenerative disorders and cancer [164]. Mitochondria dynamically adjust their content, undergo fusion and fission, and activate the unfolded protein response to ensure cellular homeostasis and steady energy flow, crucial for cellular signaling under stress [163,164,165]. However, there remains a gap in understanding how mitochondria respond to genomic DNA damage and their impact on DNA repair and cell fate despite knowing the intricate relationship between DNA damage and mitochondrial stress [166,167]. Mitophagy, selectively targeting damaged mitochondria for degradation, is pivotal for mitochondrial quality control, primarily mediated by the phosphatase and tensin homolog-induced putative kinase 1 (PINK1)-Parkin cascade, whereby impaired mitochondria are labeled with phosphorylated ubiquitin, and then targeted for degradation. Mitophagy plays a crucial role in the early stages of Dox-induced cardiomyopathy [112,168,169]. Yet, disruptions in mitophagy can lead to a cascade of deleterious effects, ranging from metabolic disorders to cancer, senescence, inflammation, genomic instability, and aging [170]. Moreover, excessive mitophagy can be detrimental as evidenced by elevated LC3 and Beclin 1 levels, decreased p62 expression during Dox treatment, and suppressed expression of PGC-1α, NRF1, and TFAM, along with reductions in mitochondrial protein levels. Furthermore, Dox induces concentration-dependent reductions in mitochondrial membrane potential (MMP) and mtDNA content [171]. Notably, cells treated with Dox exhibit significant MMP reductions to 55% of the control and mtDNA content declines up to 83% of the control at specific Dox doses [171]. Interestingly, recent insights suggest a potential involvement of DNA repair proteins such as FANCD2, BRCA1, and BRCA2 not only in repairing nuclear DNA but also in Parkin-mediated mitophagy [172]. Additionally, the presence of HR machinery within mitochondria, supported by the detection of recombination intermediates and the presence of RAD51 and its related proteins like XRCC3, suggest their involvement in mitochondrial HR processes [173,174]. While key DNA damage repair proteins like BRCA1 and 53BP1 are found in mitochondria, their direct role in mitochondrial repair remains unclear [175,176]. This intriguing intersection of DNA repair BRCA1/2 proteins and mitochondrial maintenance unveils a novel dimension of cellular quality control, offering insights into therapeutic potential in mitigating Dox-induced toxicity and preserving mitochondrial health.

## 6. Autophagy Modulation for Therapeutic Intervention

Autophagy modulation holds significant therapeutic potential across diseases like cancer [177]. In cancer, autophagy’s role can either promote or inhibit tumor growth depending on context, making its therapeutic modulation complex and context-dependent [178,179]. While autophagy inhibition can sensitize certain cancer cells to chemotherapy or radiation therapy, its activation may promote tumor cell death under specific conditions [179]. Chemotherapy, a common cancer treatment, often encounters drug resistance. Autophagy aids cancer cells in surviving chemotherapy by repairing DNA and eliminating toxins, thereby contributing to drug resistance [180]. Autophagy is implicated in resistance to drugs like Dox, with research showing elevated autophagic activity in drug-resistant cells and its inhibition leading to re-sensitization to Dox [135,181]. Conversely, inhibiting autophagy can restore drug sensitivity through agents like CQ or by downregulating autophagy-associated proteins such as ATG7 and ATG14 [182,183,184,185,186]. Pharmacological inhibition of autophagy by agents like CQ and bafilomycin affects Dox efficacy. CQ potentiates Dox-induced cell death by blocking autophagic flux and reducing drug resistance in various cancers [187,188]. Bafilomycin A1, which inhibits vacuolar-type H+-ATPase and blocks lysosomal acidification, impedes autophagosome–lysosome fusion, disrupting autophagic flux crucial for cellular degradation processes, which in turn promotes the efficacy of Dox against cancer [189]. In HepG2 liver cancer cells, treatment with bafilomycin A1 or chloroquine alone at lower doses enhanced inhibition of cell growth and increased apoptosis when combined with Dox. Additionally, these inhibitors promoted lysosomal membrane permeabilization and reduced mitochondrial membrane potential in response to Dox treatment, suggesting their potential to synergistically improve Dox’s anticancer effects in liver cancer therapy [190].

In a recent study, Montalvo et al. targeted autophagy with rAAV-dnATG5 in female rats exposed to Dox and revealed that while Dox had negative effects on left ventricular function, redox balance, and mitochondrial function, acute inhibition of autophagy mitigated the increase in mitochondrial ROS emission and improved cardiac health. Notably, this improvement was observed only at the acute stage, suggesting variations in ATG5–ATG12 conjugation kinetics [191]. Natural substances like epigallocatechin-3-gallate (EGCG) and magnoflorine have also been explored for enhancing Dox efficacy by regulating autophagy [134,192]. Another compound, metformin, a remarkable antidiabetic medication, not only regulates blood sugar levels but also holds tremendous promise in cancer therapy through its intricate modulation of cellular processes [193]. By activating AMPK, metformin elevates the AMPK/AMP ratio, setting off a cascade of events. AMPK activation, in turn, exerts its inhibitory influence on the mechanistic target of rapamycin complex 1 (mTORC1) pathway by phosphorylating key downstream targets like tuberous sclerosis complex protein 2 (TSC2) [194]. This inhibition of mTORC1 serves as a crucial checkpoint, curbing aberrant cell growth and proliferation, hallmarks of cancer [195]. Moreover, metformin’s impact extends to the modulation of autophagy, the cellular process of self-degradation and recycling. Through its influence on AMPK and mTORC1, metformin enhances autophagic activity, promoting the selective degradation of damaged proteins and organelles within cancer cells [196].

Studies also investigated whether altering autophagy could mitigate Dox-induced cardiotoxicity. Accordingly, rat cardiac myoblasts were treated with rapamycin, an mTOR inhibitor and autophagy inducer, prior to Dox exposure, which showed significantly improved cell viability, reduced apoptosis and ROS production, and enhanced mitochondrial function. To further validate findings, they used GFP-LC3 mice with EO771 tumors, where a single rapamycin injection followed by Dox injections preserved cardiac function, as evidenced by reduced caspase-3 expression, and maintained cardiomyocyte size [197]. Rapamycin’s autophagy induction potentially protects against Dox cardiotoxicity by promoting cellular survival mechanisms. Conversely, rapamycin complicates the therapeutic landscape by potentially promoting cancer cell survival against Dox-induced cytotoxicity. Recent research suggests that rapamycin-mediated autophagy induction might confer resistance to Dox in certain cancer contexts, highlighting the dual-edge sword of autophagy modulation in cancer therapy warranting future investigations [198]. Other agents, like thymoquinone (TQ), induced autophagy in Dox-treated cardiac myoblasts by activating LKB1/AMPK and inhibiting mTOR, and promoted cardiac myoblasts survival. Blocking autophagosome formation reversed TQ’s anti-apoptotic effects, suggesting its potential to prevent Dox-induced cardiotoxicity [199]. Another drug Spinacetin is proposed to protect cardiac myoblasts against Dox-induced cytotoxicity by enhancing autophagy and reducing apoptosis via AMPK/mTOR signaling that is mediated by increased SIRT3 expression [200]. Furthermore, curcumin induces autophagy in various tumor cells by suppressing the PI3K/Akt/mTOR pathway and enhancing LC3-II, Beclin1, and ATG protein expression. Studies in ovarian, gastric, and lung cancer cells highlight curcumin’s role in promoting autophagic vesicle formation and reducing p62 levels, potentially enhancing cancer treatment efficacy through autophagy modulation [201,202]. Separately, Zhang et al. and Wei Yu et al. independently demonstrated in their study that curcumin attenuates Dox-induced cardiotoxicity in mice [203,204]. These findings suggest curcumin’s potential as a protective agent against Dox-induced cardiotoxicity while illustrating its complex role in modulating autophagy in different contexts. Understanding the context-specific effects of these compounds on autophagy could promote precision medicine.

Precision medicine is mainly driven by omic technologies where, by analyzing genetic variants, protein expression patterns, and metabolic profiles, clinicians can stratify patients based on their individual molecular signatures. This personalized approach allows for tailored treatment strategies that maximize efficacy while minimizing adverse effects. Proteomic analysis enables the characterization of protein expression changes induced by autophagy modulation, identifying biomarkers associated with autophagy activity or response to specific drugs. For instance, biomarkers like the LC3-II/I ratio, reflecting autophagic flux, have been extensively studied in cancer to predict treatment efficacy. Elevated LC3-II levels indicate increased autophagy and may predict resistance to chemotherapy in certain cancer types [205]. Conversely, p62/SQSTM1 levels, which accumulate when autophagy is impaired, have been linked to poor prognosis in various cancers [206]. Proteomic techniques have pinpointed specific proteins like lysosomal-associated membrane protein 2 (LAMP2A) and serine/threonine kinase 11 interacting protein (STK11IP), crucial for regulating lysosomal targeting and mTORC1 signaling in diseases such as cancer [207,208]. These studies underscore how dynamic changes in autophagy-related protein expression influence disease pathogenesis, which can be monitored and used by clinicians to predict treatment responses [205]. By incorporating these biomarkers into clinical decision algorithms, physicians can tailor treatment regimens in real time, adjusting dosages or switching therapies based on individual patient responses. Genomic studies complement these insights by identifying mutations and epigenetic alterations in key autophagy genes (e.g., ATG5, ATG7), linking genetic variants to disease susceptibility and responses to treatment [209,210]. Genetic profiling has further identified polymorphisms in these genes that influence autophagy pathway dynamics and treatment responses [211]. Computational models integrate these genetic variants with clinical data to predict how patients will respond to autophagy modulation therapies. By understanding individual genetic predispositions, clinicians can adjust dosing regimens or select the most effective treatment options, thereby optimizing therapeutic outcomes. Computational models have successfully predicted treatment outcomes in cancer therapies by elucidating the complex interactions of autophagy modulation with chemotherapy drugs like Dox. For example, studies have shown that inhibiting autophagy using CQ enhances Dox’s cytotoxic effects in various cancers [187]. Computational simulations predicted the synergistic effects of combining CQ with Dox based on pharmacokinetic and pharmacodynamic data, guiding clinical decision-making [187]. In another study, computational models highlighted how rapamycin-induced autophagy protects against Dox-induced cardiotoxicity by promoting pro-survival mechanisms [197]. By simulating drug interactions within autophagy pathways, these models identify optimal dosing strategies to mitigate cardiotoxic effects while maintaining therapeutic efficacy in cancer treatment. These genetic variants can impact sensitivity to autophagy inhibitors or activators, highlighting the importance of personalized treatment approaches based on patient-specific genetic profiles. Furthermore, metabolomic profiling reveals alterations in cellular metabolism induced by autophagy modulation, providing deeper insights into its physiological consequences [212].

Moreover, integrating omics methods with advanced bioinformatics tools enhances our understanding of autophagy modulation and facilitates precision medicine approaches in treating autophagy-related disorders. Bioinformatics and computational modeling tools play a pivotal role in leveraging omics data to predict patient responses to autophagy modulation therapies. Computational models have been instrumental in studying how autophagy modulation affects treatment outcomes, particularly in cancer therapies [213,214]. Similar computational approaches have successfully elucidated complex interactions between autophagy activity and disease progression in Alzheimer’s disease and degenerative aging [215,216]. These models explore how failures in regulatory mechanisms can lead to elevated autophagy levels, potentially contributing to neuronal dysfunction through amyloid-β (Aβ) accumulation. Computational modeling approaches, exemplified by studies on Dox treatment in triple-negative breast cancer patients, provide insights into optimizing drug interactions and dosing strategies tailored to individual patient profiles, thereby advancing precision medicine in cancer therapy [217]. Future directions in this field involve refining computational models to integrate multi-omics data, including biomarkers. By combining genomics, proteomics, and metabolomics with clinical outcomes, researchers aim to develop predictive models for patient-specific responses to autophagy modulation therapies. These models will enable tailored treatment strategies based on individual genetic profiles, molecular signatures, and metabolic profiles, optimizing therapeutic efficacy while minimizing adverse effects.

## 7. Challenges and Future Directions

Translating preclinical data on autophagy-based therapies into clinical practice encounters several challenges [218]. These include potential off-target effects of pharmacological agents, limited specificity of autophagy modulators, and the necessity for biomarkers to monitor autophagic activity in patients [219]. Furthermore, the complexity of autophagy regulation and its context-dependent effects necessitate further research to optimize therapeutic strategies and identify patient populations most likely to benefit from autophagy modulation [179]. It is noteworthy that a transient surge in autophagy signaling post-Dox exposure cautions against prolonged autophagy inhibition due to potential pathological ramifications [191]. Thus, future research avenues are warranted to fine-tune autophagy modulation in therapeutic interventions. While both augmentation and suppression of autophagy have been proposed as potential approaches, the current clinical landscape predominantly leans toward inhibiting autophagy [182,220]. This inclination underscores a prevailing skepticism regarding the efficacy of autophagy enhancement in cancer therapy. However, as clinical trials explore this intricate interplay, the decision to predominantly target autophagy inhibition signals a cautious yet determined stride toward unraveling the complexities of cancer treatment. The relationship between autophagy and Dox in cancer treatment is complex and multifaceted [221]. Depending on numerous variables, including cancer type, the patient’s genetic background, and treatment circumstances, autophagy may either shield cancer cells from Dox-induced cytotoxic effects or promote cell death. Understanding these mechanisms is pivotal for developing effective therapeutic approaches that optimize autophagy regulation to enhance Dox’s efficacy in cancer therapy. Additionally, recent studies suggest that BRCA1/BRCA2, well-known tumor suppressor genes frequently mutated in hereditary breast and ovarian cancers, may play a role in autophagy regulation. BRCA1 has been implicated in promoting autophagy under cellular stress conditions, while BRCA2 deficiency has been associated with impaired autophagy flux, potentially contributing to cancer development and treatment resistance [138,159]. Investigating the interplay and molecular mechanism between BRCA1/BRCA2 and autophagy could provide further insights into cancer biology and therapeutic strategies promoting precision medicine. Through an in-depth investigation of the complex interaction between autophagy and Dox, researchers and medical professionals can potentially devise targeted and efficient cancer treatments, overcoming drug resistance challenges and improving patient responses to treatments.

## 8. Conclusions

The intricate interplay among autophagy, inflammation, and genomic stability underscores their collective impact on cellular health and disease processes. Autophagy, essential for removing damaged components, plays a crucial role in maintaining genomic stability. In contrast, inflammation, often triggered by DNA damage, can worsen cellular dysfunction. This complex relationship is evident in conditions like Dox-induced cardiomyopathy where DNA damage-induced inflammation activates autophagy, further influencing disease progression. Moreover, the disruption of autophagy and DNA repair mechanisms, as observed in Dox-induced cardiotoxicity, highlights their importance in determining therapeutic outcomes. The involvement of DNA damage repair genes, particularly BRCA1/2 emphasizes their critical role in mediating DNA repair, autophagy, and disease susceptibility. However, the exact mechanisms through which these genes regulate autophagy remain unclear, presenting a crucial area for further investigation. Additionally, mitophagy emerges as a pivotal mechanism in maintaining mitochondrial quality control and cellular homeostasis, with implications for disease development and therapeutic interventions. From a therapeutic standpoint, modulating autophagy shows promise for various diseases, including cancer. However, translating autophagy-based therapies into clinical practice faces challenges, such as off-target effects and the limited specificity of pharmacological agents. Further research is necessary to refine therapeutic strategies and identify patient populations who are most likely to benefit from autophagy modulation. Ultimately, unraveling the complex interplay among autophagy, inflammation, and genomic stability will lead to the development of novel therapeutic interventions and personalized medicine approaches.
